# Structural and Functional Trajectories of Middle Temporal Gyrus Sub-Regions During Life Span: A Potential Biomarker of Brain Development and Aging

**DOI:** 10.3389/fnagi.2022.799260

**Published:** 2022-04-27

**Authors:** Jinping Xu, Jinhuan Zhang, Jiaying Li, Haoyu Wang, Jianxiang Chen, Hanqing Lyu, Qingmao Hu

**Affiliations:** ^1^Institute of Biomedical and Health Engineering, Shenzhen Institute of Advanced Technology, Chinese Academy of Sciences, Shenzhen, China; ^2^Department of Radiology, Shenzhen Traditional Chinese Medicine Hospital, The Fourth Clinical Medical College, Guangzhou University of Chinese Medicine, Shenzhen, China; ^3^School of Artificial Intelligence, University of Chinese Academy of Sciences, Beijing, China; ^4^CAS Key Laboratory of Human-Machine Intelligence-Synergy Systems, Shenzhen Institute of Advanced Technology, Chinese Academy of Sciences, Shenzhen, China

**Keywords:** middle temporal gyrus, gray matter volume, resting-state functional connectivity, regression model, deep learning network

## Abstract

Although previous studies identified a similar topography pattern of structural and functional delineations in human middle temporal gyrus (MTG) using healthy adults, trajectories of MTG sub-regions across lifespan remain largely unknown. Herein, we examined gray matter volume (GMV) and resting-state functional connectivity (RSFC) using datasets from the Nathan Kline Institute (NKI), and aimed to (1) investigate structural and functional trajectories of MTG sub-regions across the lifespan; and (2) assess whether these features can be used as biomarkers to predict individual’s chronological age. As a result, GMV of all MTG sub-regions followed U-shaped trajectories with extreme age around the sixth decade. The RSFC between MTG sub-regions and many cortical brain regions showed inversed U-shaped trajectories, whereas RSFC between MTG sub-regions and sub-cortical regions/cerebellum showed U-shaped way, with extreme age about 20 years earlier than those of GMV. Moreover, GMV and RSFC of MTG sub-regions could be served as useful features to predict individual age with high estimation accuracy. Together, these results not only provided novel insights into the dynamic process of structural and functional roles of MTG sub-regions across the lifespan, but also served as useful biomarkers to age prediction.

## Introduction

The middle temporal gyrus (MTG) is suggested to be involved in various functions (Giraud et al., 2004; Sato et al., 2012) with several distinct sub-regions (Sewards, 2011). Recently, a consistent topography pattern of structural and functional delineations in MTG was identified in our previous studies (Xu et al., 2015, 2019a), resulting in four distinct clusters, namely, the anterior part of MTG (aMTG), the middle part of MTG (mMTG), the posterior part of MTG (pMTG), and the sulcus part of MTG (sMTG). Although structural and functional roles of MTG were decoded in the healthy adults (21–25 years), accumulating evidence of significant age-related effects on structural and functional patterns of MTG was identified. Previous studies based on functional MRI (Blumenfeld et al., 2006; Chou et al., 2006) showed age-related brain activation in left MTG, whereas one similar study showed no age effect (Moore-Parks et al., 2010). Using other functional neuroimaging technologies, such as magnetoencephalography, old adults exhibited both declined cortical activities and delayed activation in MTG as compared to younger adults (Lin et al., 2018). Moreover, two graph theory studies (Cao et al., 2014; Zhao et al., 2015) also showed inversed U-shaped trajectories of nodal degree and functional connectivity strength in the MTG across the lifespan (7–85 years). Structurally, the thickness of left MTG showed a negative relationship with age (10–60 years) (Raznahan et al., 2010), and the gray matter volume (GMV) of MTG showed a dramatic decline from 6 to 26 years (Mu et al., 2017). In addition to healthy individuals, patients with reading impairment presented a delay in the development of left MTG in the children group (Luniewska et al., 2019). Moreover, our recent study revealed specific structural and functional patterns of MTG sub-regions in children and adults with autism spectral disorders (Xu et al., 2019b). Together, these studies provided enough evidence to indicate that the structural and functional roles of MTG are dynamic processes with remarkable changes across the lifespan (Fair et al., 2008; Zhao et al., 2015; Li et al., 2019). Thus, investigating age-related trajectories of MTG sub-regions across the lifespan is critical and helpful to guide the interpretation of alterations associated with these regions in many brain disorders.

To date, many studies have been performed to investigate lifespan trajectory about gray matter (Gennatas et al., 2017; Mu et al., 2017), white matter (Ziegler et al., 2012; Zhao et al., 2015), cortical morphology (Sowell et al., 2003; Khundrakpam et al., 2015), and topography of structural and functional brain networks (Fair et al., 2008; Betzel et al., 2014; Li et al., 2019), revealing that the structure and function of the human brain undergo complex changes across the lifespan. Moreover, several studies have been performed to characterize age-related effects on functional connectome (Wang et al., 2012; Cao et al., 2014; Yang et al., 2014; Jiang et al., 2019) using the same dataset (NKI sample). For example, Cao et al. (2014) observed that the human brain functional connectome not only exhibited highly preserved nonrandom modular organization, but also showed linearly decreases in modularity, inverted-U–shaped trajectories of local efficiency, and inverted-U–shaped trajectories of rich club architecture over the entire age range. Wang et al. (2012) showed a linear increase in the emotion system and a decrease in the sensorimotor system during brain development from childhood to senescence. Yang et al. (2014) observed stronger age dependence in the spatial pattern of a precuneus dorsal–posterior cingulate cortex network as compared to the default network. However, little attention was paid to the lifespan changes of particular regions at the sub-regional level. Therefore, our study aimed to investigate the structural and functional lifespan trajectory of MTG at the sub-regional level in healthy individuals.

Moreover, age prediction is considered to be an important way to understand brain developmental and degenerative processes in healthy individuals (Zhao et al., 2019). Many brain structural and functional features are sensitive to maturational processes throughout the lifespan, such as cortical thickness (Khundrakpam et al., 2015; Lewis et al., 2018), GMV (Franke et al., 2010; Mohajer et al., 2020), resting-state functional connectivity (RSFC) (Ciesielski et al., 2019; Li et al., 2019), brain network topography (Cao et al., 2014; Zhao et al., 2015), as well as multi-modal MRI features (Betzel et al., 2014; Zhao et al., 2019). However, these studies mainly focused on extracting different features to increase the accuracy of age prediction using traditional machine learning techniques (i.e., non-deep learning algorithms) rather than improving prediction models. Recently, the Long Short-Term Memory (LSTM) deep learning model (Hochreiter and Schmidhuber, 1997), an evolution over the Recurrent Neural Networks (RNN), has been widely adopted in clinical predictions, such as epileptic seizures (Tsiouris et al., 2018; Wei et al., 2019), heart failure (Maragatham and Devi, 2019), cancer survival outcomes (Koo et al., 2020), and even mortality of patients (Xia et al., 2019). This method succeeded better performance than traditional ways (Tsiouris et al., 2018; Wei et al., 2019; Xia et al., 2019), suggesting potential applications to increase the accuracy of age prediction.

In the current study, we aimed to (1) investigate structural and functional trajectories of MTG sub-regions across the lifespan; and (2) assess whether these features can be used as biomarkers to accurately predict an individual’s chronological age. To uncover these two questions, we used T1 structural and resting-state fMRI data from the publicly available Nathan Kline Institute-Rockland Sample (NKI-RS) dataset (7–85 years)^1^. The GMV and RSFC patterns of each MTG sub-regions were calculated and subsequently used to explore their relationship with age using linear and quadric regression models. Finally, the structural and functional features of MTG were used as inputs for LSTM to predict an individual’s chronological age.

## Materials and Methods

### Participants

Data used in this study are publicly available at the International Neuroimaging Data-sharing Initiative (INDI) (see text footnote 1) from the Nathan Kline Institute (NKI, NY, United States). The initial dataset included 207 participants. The inclusion criteria were (1) aged 7–85 years; (2) without any lesions in brain scans; (3) no problems in semi-structured diagnostic psychiatric interviews; and (4) normal in psychiatric assessments. We also excluded participants who were (1) left-handed; (2) missing demographic data; (3) with bad image integrity and quality on screening; and (4) only T1 or fMRI images. Finally, 160 subjects were included in the structural analyses and functional analyses. These subjects include 25 children and adolescents (10 female / 15 male, 22 right / 3 left-handedness, age range of 7–18 years), 48 young adults (22 female / 26 male, 43 right / 5 left-handedness, age range of 19–30 years), 37 middle-aged adults (11 female / 26 male, 31 right / 6 left-handedness, age range of 31–45 years), 22 older adults (7 female / 15 male, 20 right / 2 left-handedness, age range of 46–60 years), and 28 elders (12 female / 16 male, 24 right / 4 left-handedness, age range of 61–85 years). This information is given in Supplementary Table 1. Informed consent was obtained from all subjects, and the study was approved by the NKI institutional review board (Nooner et al., 2012). Moreover, this study was performed in accordance with the Declaration of Helsinki.

### MRI Acquisition

Magnetic resonance imaging scanning was performed using a Siemens 3.0 T Trio Tim MRI scanner. During scanning, subjects were told to keep their eyes closed, relax their minds, and to not move. T1 images were obtained using the magnetization-prepared rapid gradient echo (MPRAGE) sequence: time repetition (TR) / time echo (TE) = 2,500 / 3.5 ms, inversion time = 1,200 ms, flip angle (FA) = 8°, field of view (FOV) = 256 mm × 256 mm, voxel size = 1.0 mm × 1.0 mm × 1.0 mm, and number of slices = 192. Resting-state fMRI scans were collected using an echo-planar imaging (EPI) sequence: TR / TE = 2500 / 30 ms, FA = 80°, FOV = 216 mm × 216 mm, voxel size = 3.0 mm × 3.0 mm × 3.0 mm, number of slices = 38, and 260 volumes.

### Image Preprocessing

The T1 images were preprocessed using a toolbox for Data Processing & Analysis for Brain Imaging (DPABI)^2^. First, the quality of each image was visually checked and 30 subjects were excluded for really bad quality. Then, the remaining T1 images were segmented into gray matter, white matter, and cerebrospinal fluid, and transformed to a standard Montreal Neurological Institute space. Next, these images were modulated to preserve regional volume information. Finally, the modulated gray matter images were smoothed with a Gaussian kernel of 6 mm full-width at half maximum and used in the following analyses.

The resting-state fMRI data were also preprocessed using DPABI. The main steps included the following: (1) removing the first 10 volumes; (2) slice timing; (3) realigning (subjects with head motion exceeding 3 mm in any dimension or 3° of angular motion were removed, and 20 subjects were excluded); (4) spatially normalizing; (5) resampling to a voxel size of 3 mm × 3 mm × 3 mm; (6) smoothing using a Gaussian kernel of 6 mm full-width at half maximum; (7) removing linear and quadratic trends; (8) regressing out head motion effects using the Friston 24-parameter model (Satterthwaite et al., 2013), as well as the white matter, cerebrospinal fluid, and global signals; (9) temporal band-pass filtering (0.01–0.1 Hz); and (10) “scrubbed” two-time points before and one time point after bad images, whose frame displacement (FD) > 0.5 (Power et al., 2012).

### Definition of Middle Temporal Gyrus Sub-Regions

The MTG sub-regions were obtained from our previous results (Xu et al., 2019a), which were defined as the overlap between functional and anatomical parcellation results of MTG (Supplementary Figure 1). All the MTG sub-regions were then resampled to 1.5 mm × 1.5 mm × 1.5 mm for structural analyses and 3 mm × 3 mm × 3 mm for RSFC analyses.

### Calculations of Structural and Functional Indexes

Structurally, the GMV was defined as the mean value of each MTG sub-region in the modulated and smoothed gray matter map. The analysis of covariance (ANCOVA) was performed to identify between-group differences. The significant levels were set at *P* < 0.05 with Bonferroni correction.

The RSFC was defined as the Pearson correlation coefficients between the mean time series of each MTG sub-region and that of each voxel in the rest of the brain, respectively. Then, all correlation coefficients were converted to *z* values using Fisher’s *z* transformation to improve normality.

### Age Effects on Regional Gray Matter Volume

To determine how age affects GMV of MTG sub-regions, both linear and quadric regression models were used controlling for covariates of gender and handedness. The significant level was set at *p* < 0.05/8 = 0.00625. The models for GMV can be expressed with the following equations:


Y=β+0β×1age+β×2gender+β×3handedness(linear);

Y=β+0β×1age+β×2age+2β×3gender+

Then, Akaike’s information criterion (AIC) was used to determine the best-fitting model. The extreme age along the quadric trajectory is important to the investigation of timing differences in maturation or degeneration across the lifespan, and could be calculated using age = -β_1_/2β_2._ The fitted GMV was defined as the residual of GMV regressed out gender, handedness, and age.

### Age Effects on Resting-State Functional Connectivity

To explore the corresponding quadric relationship between age and RSFC of MTG sub-regions, we performed correlation analyses in the DPABI using age^2^ as an independent variable, and gender, handedness, age, and FD as covariates. The significance level was determined using the Gaussian random field corrections with a voxel-level threshold of *p* < 0.001 and a cluster-level threshold of *p* < 0.05.

For the regions which showed a significant quadric relationship with age, we calculated their mean RSFC and performed quadric regression models controlling for covariates of gender, handedness, and FD. The model can be expressed by: Y = β_0_ + β_1_ × age + β_2_ × age^2^ + β_3_ × gender + β_4_ × handedness + β_5_ × FD. The significant level was set at *p* < 0.05/15 = 0.003. The extreme age was also calculated using Age = -β_1_/2β_2_. The fitted RSFC was defined as the residual of RSFC regressed out the gender, handedness, age, and FD.

### Long Short-Term Memory Network to Predict Individual Age

We adopted a commonly used LSTM network (Tsiouris et al., 2018; Liu and Gong, 2019; Supplementary Figure 2) to predict individual age using 23 features, including GMV of all MTG sub-regions and all RSFC listed in Table 1. All features are standardized to 0–1 and served as inputs for a simple 4-layer LSTM network with 40 hidden units. Each hidden unit has self-memory and was fully connected. Especially, it is composed of an input gate *i*, an output gate *h*, and a forgetting gate *f*, which were defined by the equations in the right bottom of Figure 2, respectively. The output layer was composed of a sigmoid function. In the initial model, the batch size was set to 10, and the training rate was set to 0.006 for the Adaptive Moment Estimation (Adam) optimizer, which used the Root-Mean-Square Error (RMSE = 1m∑i=1m(yi-yi^)2,m=1,2n) as loss function. Moreover, we used leave-one-out cross-validations to evaluate the model by 1,000 times. To assess prediction accuracy, the Pearson correlation coefficient and the Mean Absolute Error (MAE) between the actual and predicted ages were calculated. The LSTM networks were built using Keras 2.2.4 upon Tensorflow 1.12.0 backend in Python 3.6.

**TABLE 1:** Brain regions that showed a quadratic relationship between RSFC of MTG sub-regions and age.

Seed regions	Brain regions	Abbreviations	Cluster size	Peak coordinates	Peak intensity
aMTG.L	Left medial frontal gyrus	MFG.L	112	(−18, 21, −6)	0.36222
	Right vermis	VER.R	111	(6, −45, −3)	0.3867
mMTG.L	Right operculum part of inferior frontal gyrus	operIFG.R	89	(36, 12, 27)	–0.3259
sMTG.L	Left anterior cingulate cortex	ACC.L	82	(−12, 30, −9)	0.3710
	Right amygdala	AMG.R	310	(27, −6, −15)	0.3778
	Left putamen	PUT.L	292	(−27, 0, −9)	0.3893
	Right postcentral gyrus	PostCG.R	249	(36, −15, 36)	–0.3649
	Left postcentral gyrus	PostCG.L	296	(−39, −12, 33)	–0.3757
aMTG.R	Right pole part of superior temporal gyrus	pSTG.R	54	(42, 18, −24)	0.3443
pMTG.R	Right superior temporal gyrus	STG.R	46	(51, −21, 0)	0.3521
	Left caudate	CAU.L	52	(−15, 9, 12)	0.3522
sMTG.R	Right pallidum	PAL.R	211	(21, −6, −6)	0.3769
	Left anterior cingulate cortex	ACC.L	150	(−9, 33, 0)	0.3408
	Left precentral gyrus	PreCG.L	1038	(−36, −18, 51)	–0.4014
	Right precentral gyrus	PreCG.R	516	(60, −9, 45)	–0.3964

## Results

### Age Effects on Regional Gray Matter Volume

**FIGURE 1:**
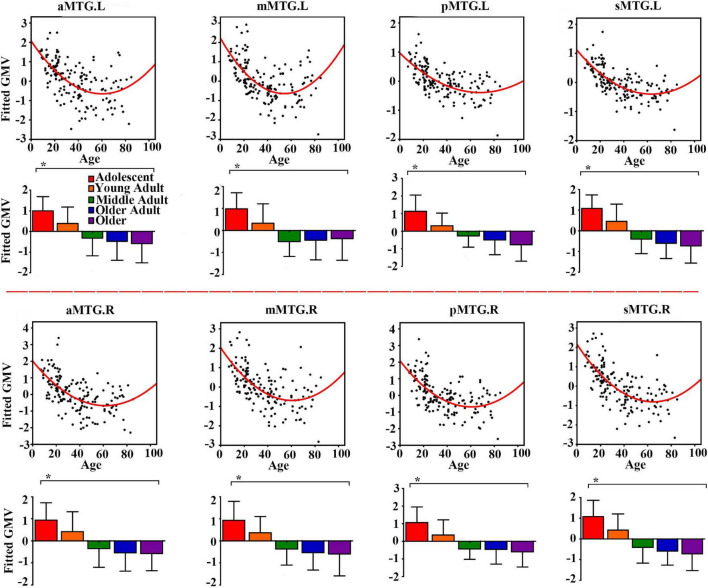
Quadratic relationships between gray matter volume (GMV) of middle temporal gyrus (MTG) sub-regions and age across lifespan. The GMV was fitted by controlling for gender and handedness. The analysis of covariance (ANCOVA) was performed to explore group differences (* represents significant difference).

Controlling for effects of gender and handedness, the fitted GMV of all MTG sub-regions exhibited U-shaped trajectories with troughs nearly 60 years (Figure 1).

### Age Effects on Resting-State Functional Connectivity

**FIGURE 2:**
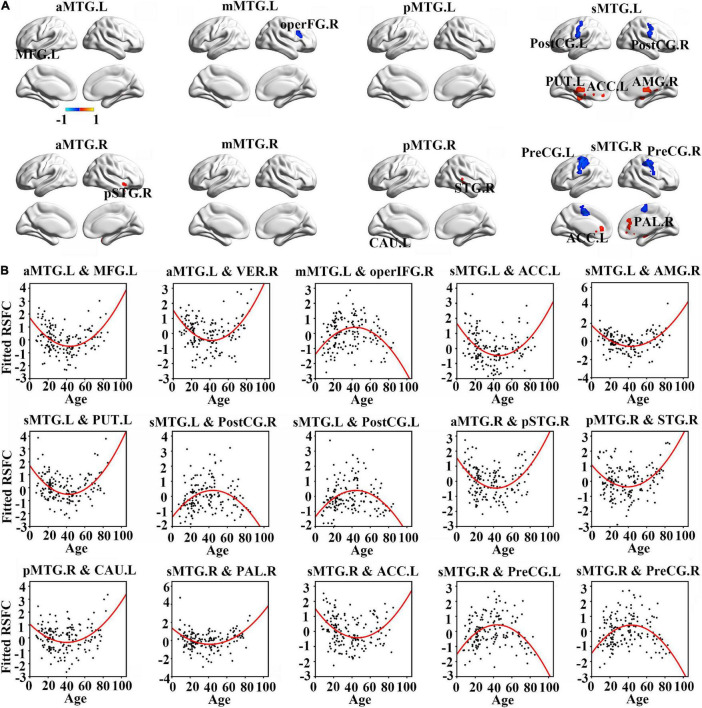
**(A)** Brain regions which showed a quadratic relationship between resting-state functional connectivity (RSFC) of MTG sub-regions and age across lifespan. **(B)** Quadratic relationships between RSFC of MTG sub-regions and age across lifespan. Abbreviations of brain regions are listed in Table 1.

Resting-state functional connectivity between MTG sub-regions and most cortical brain regions showed inversed quadric relationships with age, but RSFC between MTG sub-regions and sub-cortical brain regions/cerebellum showed the opposite way (Figure 2A and Table 1).

Further quadric regression models showed significantly U-shaped or inverse U-shaped trajectories between fitted RSFC of MTG sub-regions and age with extreme age nearly 40 years (Figure 2B). Especially, RSFC between aMTG.L and left medial frontal gyrus (MFG.L)/ right vermis (VER.R) exhibited U-shaped trajectories with age. RSFC between mMTG.L and right operculum part of inferior frontal gyrus (operFG.R) showed inversed U-shaped trajectory with age. RSFC between sMTG.L and left anterior cingulate cortex (ACC.L)/ left putamen (PUT.L)/ right amygdala (AMG.R) showed U-shaped trajectories, whereas RSFC between sMTG.L and left post-central gyrus (PostCG.L)/ right post-central gyrus (PostCG.R) showed inverse way.

Resting-state functional connectivity between aMTG.R and right pole part of temporal gyrus (pSTG.R) exhibited U-shaped trajectory with age. RSFC between pMTG.R and left caudate (CAU.L)/ right superior temporal gyrus (STG.R) showed U-shaped trajectories with age. RSFC between sMTG.R and ACC.L /right pallidum (PAL.R) showed U-shaped trajectories, whereas RSFC between sMTG.R and left precentral gyrus (PreCG.L)/ right precentral gyrus (PreCG.R) showed inverse way.

### Maturation Age

**FIGURE 3:**
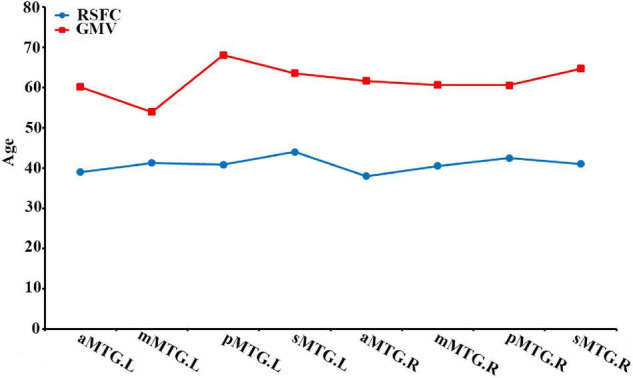
Maturation ages of RSFC and GMV in the MTG sub-regions.

We also calculated the extreme age and identified that the maturation ages of RSFC in all MTG sub-regions were nearly 20 years earlier than those of GMV (Figure 3).

### Age Predictions

**FIGURE 4:**
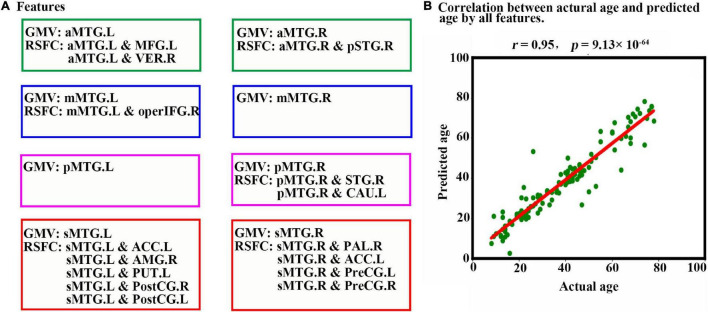
Prediction results based on features from MTG sub-regions using LSTM. **(A)** Features are used to predict an individual’s age in the LSTM network. The scatter plot depicts actual age versus predicted age by all features. **(B)** Pearson correlation analyses between the actual and predicted ages are performed to evaluate the prediction accuracy. Abbreviations of brain regions are listed in Table 1.

When all 23 features (Figure 4A) were combined as input for the LSTM network to predict individual age, a high estimation accuracy was obtained (MAE = 3.89, *r* = 0.95, *p* = 9.13 × 10^–64^) between predicted versus actual ages (Figure 4B).

## Discussion

In the current study, we (1) examined GMV and RSFC of each MTG sub-region; (2) performed both linear and quadric regression models to investigate structural and functional trajectories of MTG sub-regions across the lifespan, as well as ANCOVA to examine between-group differences; and (3) adopted LSTM to assess whether these features can be used to accurately predict an individual’s chronological age. As a result, we identified that GMV of all MTG sub-regions showed U-shaped trajectories with extreme age around the sixth decade, whereas RSFC between MTG sub-regions and most cortical brain regions showed inversed U-shaped trajectories, but RSFC between MTG sub-regions and sub-cortical regions/cerebellum showed U-shaped way. Further prediction models indicated that GMV and RSFC of MTG sub-regions could be served as biomarkers to predict individual age with extremely high estimation accuracy.

Structurally, we identified that GMV of all MTG sub-regions showed U-shaped trajectories with troughs around the sixth decade, suggesting a nonlinear decline between 7 and 60 years and a slight increase after that. These trajectories were similar to one previous study, which reported significant U-shaped trajectories across 7–85 years in whole-brain total GMV (Sowell et al., 2003), as well as gray matter density in dorsal frontal and parietal association cortices. Moreover, some developmental neuroimaging studies also supported our results by showing modest decreases in GMV in almost all cortical regions during adolescence (Sowell et al., 2004; Brain Development Cooperative Group, 2012; Gennatas et al., 2017), which is considered to be generally attributed to a combination of synaptic pruning of exuberant connections and increasing myelination, two essential aspects of cortical development (Stiles and Jernigan, 2010). However, different brain structures showed unique trajectories across the lifespan. For example, the basal ganglia generally follow linear decreases of volume over the age of 4–18 years (Brain Development Cooperative Group, 2012), 8–30 years (Ostby et al., 2009), 18–94 years (Fjell et al., 2013), as well as in a longitudinal study over the age of 22 years (Tamnes et al., 2013), whereas hippocampus indicates an inverted U exponential volumetric trajectory from childhood to puberty, peaking at a later age than the basal ganglia (Wierenga et al., 2014). Moreover, an entire lifespan study reported that the absolute global GMV follows a complex trajectory with four phases: (1) rapid increase from 0 to 8–10 years, (2) rapid decrease until 40 years, (3) a plateau from 40 to 80 years, and (4) a rapid decrease after 80 years (Coupe et al., 2017). The rapid decrease from 8 to 40 years is similar to the trajectory of MTG sub-regions to some extent. However, this cannot be used as direct evidence to support our results for the following two reasons: (1) trajectory of maturational and aging effects varied considerably over the brain cortex, with an earlier maturation of sensory, motor, and limbic regions relative to association regions (Sowell et al., 2003; Gogtay et al., 2004; Shaw et al., 2008); and (2) the gray matter reductions were markedly greater than the subcortical changes (Sullivan et al., 2011; Tamnes et al., 2013). Anyway, our results of U-shaped trajectories of MTG sub-regions extended their findings and offered detailed information for MTG maturations at the sub-regional level, which might be helpful to guide interpretation of alterations associated with these regions in many brain disorders.

Functionally, we also identified that RSFC between MTG sub-regions and cortical brain regions showed inversed U-shaped trajectories, but RSFC between MTG sub-regions and sub-cortical regions/cerebellum showed U-shaped way. These results were supported by previous findings of the rewiring and pruning of subcortical–cortical connectivity accompanied by increased cortico-cortical connectivity during development (Supekar et al., 2009), as well as increased correlation strength of subcortical–cortical connectivity but decreased cortico-cortical connectivity during aging (Tomasi and Volkow, 2012). Moreover, these results were also similar to one previous study based on fMRI, which showed inverse U-shaped trajectories between regional nodal properties for cortical regions (e.g., MTG.L, PreCG.L) and age (7–85 years) but U-shaped way for subcortical regions (e.g., HIP.L) (Cao et al., 2014). Interestingly, the maturation ages of RSFC were about 20 years earlier than those of GMV, with about 40 years. On one hand, this aligns well with the finding of age-related U-shaped trajectory of the normalized rich-club coefficient, as well as functional connectivity strength and proportions, with maturation age of approximately 40 years (Cao et al., 2014). On the other hand, our results further supported the suggestion that development and degeneration of structural and functional connectivity is not synchronous (Horn et al., 2014).

To our knowledge, it is the first illustration that LSTM networks can be used to effectively estimate chronological age based on MRI features although it has been widely adopted in other clinical predictions (Wei et al., 2019; Koo et al., 2020). As expected, our trained LSTM model showed relatively higher accuracy in age prediction with a correlation between chronological and predicted age of *r* = 0.95 (MEA = 3.89, *p* = 9.13 × 10^–64^). This performance was superior to most previous studies using traditional machine learning methods, such as support vector regression (Wang et al., 2012; Erus et al., 2015; Zhao et al., 2015), least absolute shrinkage and selection operator (Varikuti et al., 2018), kernel regression methods (Franke et al., 2012), ridge regression model (Zhao et al., 2019), and elastic net penalized linear regression model (Khundrakpam et al., 2015; Lewis et al., 2018), among which the best performance was *r* = 0.93. Other deep learning methods using convolutional neural network based on the gray matter (Wang et al., 2019) and raw imaging data (Cole et al., 2017) resulted in slightly worse performances of MAE = 4.45, and MAE = 4.16, respectively, in age predictions. Comparable to our results, a neuroanatomical model, including 231 variables derived from multiple imaging modalities, resulted in a correlation of *r* = 0.961 between chronological and predicted age and a mean prediction error across all ages of just 1.03 years (Brown et al., 2012). However, this study included typically developing individuals between 3 and 20 years rather than across lifespan (7–85 years) and more variables as compared to ours. Moreover, our results indicated that GMV and RSFC of MTG sub-regions could be served as biomarkers to predict individual age. Similar to our results, many previous studies confirmed that the whole-brain GMV (Cole et al., 2017; Varikuti et al., 2018; Wang et al., 2019) and RSFC (Wang et al., 2012), as well as multi-modal MRI features (Franke et al., 2012), were sensitive to age predictions. However, these features were based on the whole-brain level. Our results further extended previous studies by identifying features to particular brain regions at the sub-regional level.

Several limitations should be addressed. First, only GMV and RSFC were examined in the current study, additional future studies using other neuroimaging techniques, such as diffusion tensor imaging, to evaluate the lifespan trajectory of white matter fibers originated from MTG sub-regions are warranted. Second, no behavioral variables were included to measure cognitive and language abilities, which were two important functions associated with MTG (Whitney et al., 2011). Thus, brain behavior associations are missing, weakening the interpretation of our results. Finally, we only included participants from 7 to 85 years of age, investigating earlier developmental changes during infancy and early childhood is also significantly important.

## Conclusion

We identified specific trajectories for GMV and RSFC of MTG sub-regions across the lifespan. These results not only offered detailed information for MTG maturations and degeneration at the sub-regional level but also might be helpful to guide the interpretation of alterations associated with these regions in many brain disorders. Finally, LSTM networks based on GMV and RSFC of MTG sub-regions can accurately predict chronological age in healthy individuals. This can be achieved using features of particular brain regions, substantially reducing computation complexity to extract features from the whole-brain level.

## Data Availability Statement

Data used in this study are publicly available at the International Neuroimaging Data-sharing Initiative (INDI) (http://fcon_1000.projects.nitrc.org/indi/pro/nki.html#LastRelease) from the Nathan Kline Institute (NKI, NY, United States).

## Ethics Statement

The studies involving human participants were reviewed and approved by NKI institutional review board. Written informed consent to participate in this study was provided by the participants’ legal guardian/next of kin.

## Author Contributions

JX, QH, and HL designed the study. JZ and JC downloaded the data. JX, JZ, JL, and HW analyzed the data. JX wrote the manuscript. All authors reviewed the manuscript.

## Conflict of Interest

The authors declare that the research was conducted in the absence of any commercial or financial relationships that could be construed as a potential conflict of interest.

## Publisher’s Note

All claims expressed in this article are solely those of the authors and do not necessarily represent those of their affiliated organizations, or those of the publisher, the editors and the reviewers. Any product that may be evaluated in this article, or claim that may be made by its manufacturer, is not guaranteed or endorsed by the publisher.

## Footnotes

1
http://fcon_1000.projects.nitrc.org/indi/pro/nki.html#LastRelease

2
http://rfmri.org/dpabi

## References

[B1] Betzel R. F. Byrge L. He Y. Goni J. Zuo X. N. Sporns O. (2014). Changes in structural and functional connectivity among resting-state networks across the human lifespan. *Neuroimage* 102(Pt 2) 345–357. 10.1016/j.neuroimage.2014.07.067 25109530

[B2] Blumenfeld H. K. Booth J. R. Burman D. D. (2006). Differential prefrontal-temporal neural correlates of semantic processing in children. *Brain Lang* 99 226–235. 10.1016/j.bandl.2005.07.004 16098571 PMC2667123

[B3] Brain Development Cooperative Group (2012). Total and regional brain volumes in a population-based normative sample from 4 to 18 years: the NIH MRI Study of Normal Brain Development. *Cereb. Cortex* 22 1–12. 10.1093/cercor/bhr018 21613470 PMC3236790

[B4] Brown T. T. Kuperman J. M. Chung Y. Erhart M. McCabe C. Hagler D. J. (2012). Neuroanatomical assessment of biological maturity. *Curr. Biol.* 22 1693–1698. 10.1016/j.cub.2012.07.002 22902750 PMC3461087

[B5] Cao M. Wang J. H. Dai Z. J. Cao X. Y. Jiang L. L. Fan F. M. (2014). Topological organization of the human brain functional connectome across the lifespan. *Dev. Cogn. Neurosci.* 7 76–93. 10.1016/j.dcn.2013.11.004 24333927 PMC6987957

[B6] Chou T. L. Booth J. R. Burman D. D. Bitan T. Bigio J. D. Lu D. (2006). Developmental changes in the neural correlates of semantic processing. *Neuroimage* 29 1141–1149. 10.1016/j.neuroimage.2005.09.064 16275017

[B7] Ciesielski K. T. R. Stern M. E. Diamond A. Khan S. Busa E. A. Goldsmith T. E. (2019). Maturational changes in human dorsal and ventral visual networks. *Cereb Cortex* 29 5131–5149. 10.1093/cercor/bhz053 30927361 PMC7963118

[B8] Cole J. H. Poudel R. P. K. Tsagkrasoulis D. Caan M. W. A. Steves C. Spector T. D. (2017). Predicting brain age with deep learning from raw imaging data results in a reliable and heritable biomarker. *Neuroimage* 163 115–124. 10.1016/j.neuroimage.2017.07.059 28765056

[B9] Coupe P. Catheline G. Lanuza E. Manjon J. V., and Alzheimer’s Disease Neuroimaging Initiative (2017). Towards a unified analysis of brain maturation and aging across the entire lifespan: a MRI analysis. *Hum. Brain Mapp.* 38 5501–5518. 10.1002/hbm.23743 28737295 PMC6866824

[B10] Erus G. Battapady H. Satterthwaite T. D. Hakonarson H. Gur R. E. Davatzikos C. (2015). Imaging patterns of brain development and their relationship to cognition. *Cereb Cortex* 25 1676–1684. 10.1093/cercor/bht425 24421175 PMC4428302

[B11] Fair D. A. Cohen A. L. Dosenbach N. U. Church J. A. Miezin F. M. Barch D. M. (2008). The maturing architecture of the brain’s default network. *Proc. Natl. Acad. Sci. U.S.A* 105 4028–4032. 10.1073/pnas.0800376105 18322013 PMC2268790

[B12] Fjell A. M. Westlye L. T. Grydeland H. Amlien I. Espeseth T. Reinvang I. (2013). Critical ages in the life course of the adult brain: nonlinear subcortical aging. *Neurobiol. Aging* 34 2239–2247. 10.1016/j.neurobiolaging.2013.04.006 23643484 PMC3706494

[B13] Franke K. Luders E. May A. Wilke M. Gaser C. (2012). Brain maturation: predicting individual BrainAGE in children and adolescents using structural MRI. *Neuroimage* 63 1305–1312. 10.1016/j.neuroimage.2012.08.001 22902922

[B14] Franke K. Ziegler G. Kloppel S. Gaser C., and Alzheimer’s Disease Neuroimaging Initiative (2010). Estimating the age of healthy subjects from T1-weighted MRI scans using kernel methods: exploring the influence of various parameters. *Neuroimage* 50 883–892. 10.1016/j.neuroimage.2010.01.005 20070949

[B15] Gennatas E. D. Avants B. B. Wolf D. H. Satterthwaite T. D. Ruparel K. Ciric R. (2017). Age-related effects and sex differences in gray matter density, volume, mass, and cortical thickness from childhood to young adulthood. *J. Neurosci.* 37 5065–5073. 10.1523/JNEUROSCI.3550-16.2017 28432144 PMC5444192

[B16] Giraud A. L. Kell C. Thierfelder C. Sterzer P. Russ M. O. Preibisch C. (2004). Contributions of sensory input, auditory search and verbal comprehension to cortical activity during speech processing. *Cereb Cortex* 14 247–255. 10.1093/cercor/bhg124 14754865

[B17] Gogtay N. Giedd J. N. Lusk L. Hayashi K. M. Greenstein D. Vaituzis A. C. (2004). Dynamic mapping of human cortical development during childhood through early adulthood. *Proc. Natl. Acad. Sci. U.S.A.* 101 8174–8179. 10.1073/pnas.0402680101 15148381 PMC419576

[B18] Hochreiter S. Schmidhuber J. (1997). Long short-term memory. *Neural. Comput.* 9 1735–1780. 9377276 10.1162/neco.1997.9.8.1735

[B19] Horn A. Ostwald D. Reisert M. Blankenburg F. (2014). The structural-functional connectome and the default mode network of the human brain. *Neuroimage* 102(Pt 1) 142–151. 24099851 10.1016/j.neuroimage.2013.09.069

[B20] Jiang Y. Tian Y. Wang Z. (2019). Causal interactions in human amygdala cortical networks across the lifespan. *Sci. Rep.* 9:5927. 10.1038/s41598-019-42361-0 30976115 PMC6459927

[B21] Khundrakpam B. S. Tohka J. Evans A. C., and Brain Development Cooperative Group (2015). Prediction of brain maturity based on cortical thickness at different spatial resolutions. *Neuroimage* 111 350–359. 10.1016/j.neuroimage.2015.02.046 25731999

[B22] Koo K. C. Lee K. S. Kim S. Min C. Min G. R. Lee Y. H. (2020). Long short-term memory artificial neural network model for prediction of prostate cancer survival outcomes according to initial treatment strategy: development of an online decision-making support system. *World J. Urol.* 38 2469–2476. 10.1007/s00345-020-03080-8 31925552

[B23] Lewis J. D. Evans A. C. Tohka J., Brain Development Cooperative Group, Pediatric Imaging, and Genetics Study (2018). T1 white/gray contrast as a predictor of chronological age, and an index of cognitive performance. *Neuroimage* 173 341–350. 10.1016/j.neuroimage.2018.02.050 29501876

[B24] Li R. Utevsky A. V. Huettel S. A. Braams B. R. Peters S. Crone E. A. (2019). Developmental maturation of the precuneus as a functional core of the default mode network. *J. Cogn. Neurosci.* 31 1506–1519. 10.1162/jocn_a_01426 31112473

[B25] Lin M. Y. Tseng Y. J. Cheng C. H. (2018). Age effects on spatiotemporal dynamics of response inhibition: an MEG study. *Front. Aging Neurosci.* 10:386. 10.3389/fnagi.2018.00386 30515093 PMC6255792

[B26] Liu J. Gong X. (2019). Attention mechanism enhanced LSTM with residual architecture and its application for protein-protein interaction residue pairs prediction. *BMC Bioinform.* 20:609. 10.1186/s12859-019-3199-1 31775612 PMC6882172

[B27] Luniewska M. Chyl K. Debska A. Banaszkiewicz A. Zelechowska A. Marchewka A. (2019). Children with dyslexia and familial risk for dyslexia present atypical development of the neuronal phonological network. *Front. Neurosci.* 13:1287. 10.3389/fnins.2019.01287 31849595 PMC6895138

[B28] Maragatham G. Devi S. (2019). LSTM model for prediction of heart failure in big data. *J. Med. Syst.* 43:111. 10.1007/s10916-019-1243-3 30888519

[B29] Mohajer B. Abbasi N. Mohammadi E. Khazaie H. Osorio R. S. Rosenzweig I. (2020). Gray matter volume and estimated brain age gap are not linked with sleep-disordered breathing. *Hum. Brain Mapp.* 41 3034–3044. 10.1002/hbm.24995 32239749 PMC7336142

[B30] Moore-Parks E. N. Burns E. L. Bazzill R. Levy S. Posada V. Muller R. A. (2010). An fMRI study of sentence-embedded lexical-semantic decision in children and adults. *Brain Lang.* 114 90–100. 10.1016/j.bandl.2010.03.009 20627366 PMC3630793

[B31] Mu S. H. Xu M. Duan J. X. Zhang J. Tan L. H. (2017). Localizing age-related changes in brain structure using voxel-based morphometry. *Neural. Plast* 2017:6303512. 10.1155/2017/6303512 28194282 PMC5282440

[B32] Nooner K. B. Colcombe S. J. Tobe R. H. Mennes M. Benedict M. M. Moreno A. L. (2012). The NKI-Rockland sample: a model for accelerating the pace of discovery science in psychiatry. *Front. Neurosci.* 6:152. 10.3389/fnins.2012.00152 23087608 PMC3472598

[B33] Ostby Y. Tamnes C. K. Fjell A. M. Westlye L. T. Due-Tonnessen P. Walhovd K. B. (2009). Heterogeneity in subcortical brain development: a structural magnetic resonance imaging study of brain maturation from 8 to 30 years. *J. Neurosci.* 29 11772–11782. 10.1523/JNEUROSCI.1242-09.2009 19776264 PMC6666647

[B34] Power J. D. Barnes K. A. Snyder A. Z. Schlaggar B. L. Petersen S. E. (2012). Spurious but systematic correlations in functional connectivity MRI networks arise from subject motion. *Neuroimage* 59 2142–2154. 10.1016/j.neuroimage.2011.10.018 22019881 PMC3254728

[B35] Raznahan A. Toro R. Daly E. Robertson D. Murphy C. Deeley Q. (2010). Cortical anatomy in autism spectrum disorder: an in vivo MRI study on the effect of age. *Cereb Cortex* 20 1332–1340. 10.1093/cercor/bhp198 19819933

[B36] Sato W. Toichi M. Uono S. Kochiyama T. (2012). Impaired social brain network for processing dynamic facial expressions in autism spectrum disorders. *BMC Neurosci.* 13:99. 10.1186/1471-2202-13-99 22889284 PMC3459703

[B37] Satterthwaite T. D. Elliott M. A. Gerraty R. T. Ruparel K. Loughead J. Calkins M. E. (2013). An improved framework for confound regression and filtering for control of motion artifact in the preprocessing of resting-state functional connectivity data. *Neuroimage* 64 240–256. 10.1016/j.neuroimage.2012.08.052 22926292 PMC3811142

[B38] Sewards T. V. (2011). Adolf Hopf’s 1954 myeloarchitectonic parcellation of the human temporal lobe: a review and assessment. *Brain Res. Bull.* 86 298–313. 10.1016/j.brainresbull.2011.08.010 21888952

[B39] Shaw P. Kabani N. J. Lerch J. P. Eckstrand K. Lenroot R. Gogtay N. (2008). Neurodevelopmental trajectories of the human cerebral cortex. *J. Neurosci.* 28 3586–3594. 10.1523/JNEUROSCI.5309-07.2008 18385317 PMC6671079

[B40] Sowell E. R. Peterson B. S. Thompson P. M. Welcome S. E. Henkenius A. L. Toga A. W. (2003). Mapping cortical change across the human life span. *Nat. Neurosci.* 6 309–315. 10.1038/nn1008 12548289

[B41] Sowell E. R. Thompson P. M. Toga A. W. (2004). Mapping changes in the human cortex throughout the span of life. *Neuroscientist* 10 372–392. 10.1177/1073858404263960 15271264

[B42] Stiles J. Jernigan T. L. (2010). The basics of brain development. *Neuropsychol. Rev.* 20 327–348. 10.1007/s11065-010-9148-4 21042938 PMC2989000

[B43] Sullivan E. V. Pfefferbaum A. Rohlfing T. Baker F. C. Padilla M. L. Colrain I. M. (2011). Developmental change in regional brain structure over 7 months in early adolescence: comparison of approaches for longitudinal atlas-based parcellation. *Neuroimage* 57 214–224. 10.1016/j.neuroimage.2011.04.003 21511039 PMC3101309

[B44] Supekar K. Musen M. Menon V. (2009). Development of large-scale functional brain networks in children. *PLoS Biol.* 7:e1000157. 10.1371/journal.pbio.1000157 19621066 PMC2705656

[B45] Tamnes C. K. Walhovd K. B. Dale A. M. Ostby Y. Grydeland H. Richardson G. (2013). Brain development and aging: overlapping and unique patterns of change. *Neuroimage* 68 63–74. 10.1016/j.neuroimage.2012.11.039 23246860 PMC5378867

[B46] Tomasi D. Volkow N. D. (2012). Aging and functional brain networks. *Mol. Psychiatry* 17:471. 549-58, 10.1038/mp.2011.81 PMC3193908 21727896

[B47] Tsiouris K. Pezoulas V. C. Zervakis M. Konitsiotis S. Koutsouris D. D. Fotiadis D. I. (2018). A Long Short-Term Memory deep learning network for the prediction of epileptic seizures using EEG signals. *Comput. Biol. Med.* 99 24–37. 10.1016/j.compbiomed.2018.05.019 29807250

[B48] Varikuti D. P. Genon S. Sotiras A. Schwender H. Hoffstaedter F. Patil K. R. (2018). Evaluation of non-negative matrix factorization of grey matter in age prediction. *Neuroimage* 173 394–410. 10.1016/j.neuroimage.2018.03.007 29518572 PMC5911196

[B49] Wang J. Knol M. J. Tiulpin A. Dubost F. de Bruijne M. Vernooij M. W. (2019). Gray matter age prediction as a biomarker for risk of dementia. *Proc. Natl. Acad. Sci. U.S.A.* 116 21213–21218. 10.1073/pnas.1902376116 31575746 PMC6800321

[B50] Wang L. Su L. Shen H. Hu D. (2012). Decoding lifespan changes of the human brain using resting-state functional connectivity MRI. *PLoS One* 7:e44530. 10.1371/journal.pone.0044530 22952990 PMC3431403

[B51] Wei X. Zhou L. Zhang Z. Chen Z. Zhou Y. (2019). Early prediction of epileptic seizures using a long-term recurrent convolutional network. *J. Neurosci. Methods* 327:108395. 10.1016/j.jneumeth.2019.108395 31408651

[B52] Whitney C. Jefferies E. Kircher T. (2011). Heterogeneity of the left temporal lobe in semantic representation and control: priming multiple versus single meanings of ambiguous words. *Cereb. Cortex* 21 831–844. 10.1093/cercor/bhq148 20732899 PMC3059883

[B53] Wierenga L. Langen M. Ambrosino S. van Dijk S. Oranje B. Durston S. (2014). Typical development of basal ganglia, hippocampus, amygdala and cerebellum from age 7 to 24. *Neuroimage* 96 67–72. 10.1016/j.neuroimage.2014.03.072 24705201

[B54] Xia J. Pan S. Zhu M. Cai G. Yan M. Su Q. (2019). A long short-term memory ensemble approach for improving the outcome prediction in intensive care unit. *Comput. Math Methods Med.* 2019:8152713. 10.1155/2019/8152713 31827589 PMC6885179

[B55] Xu J. Lyu H. Li T. Xu Z. Fu X. Jia F. (2019a). Delineating functional segregations of the human middle temporal gyrus with resting-state functional connectivity and coactivation patterns. *Hum. Brain Mapp.* 40 5159–5171. 10.1002/hbm.24763 31423713 PMC6865466

[B56] Xu J. Wang C. Xu Z. Li T. Chen F. Chen K. (2019b). Specific functional connectivity patterns of middle temporal gyrus subregions in children and adults with autism spectrum disorder. *Autism Res.* 13 410–422. 10.1002/aur.2239 31729198

[B57] Xu J. Wang J. Fan L. Li H. Zhang W. Hu Q. (2015). Tractography-based parcellation of the human middle temporal gyrus. *Sci. Rep.* 5:18883. 10.1038/srep18883 26689815 PMC4686935

[B58] Yang Z. Chang C. Xu T. Jiang L. Handwerker D. A. Castellanos F. X. (2014). Connectivity trajectory across lifespan differentiates the precuneus from the default network. *Neuroimage* 89 45–56. 10.1016/j.neuroimage.2013.10.039 24287438 PMC3944140

[B59] Zhao T. Cao M. Niu H. Zuo X. N. Evans A. He Y. (2015). Age-related changes in the topological organization of the white matter structural connectome across the human lifespan. *Hum. Brain Mapp.* 36 3777–3792. 10.1002/hbm.22877 26173024 PMC6869038

[B60] Zhao Y. Klein A. Castellanos F. X. Milham M. P. (2019). Brain age prediction: cortical and subcortical shape covariation in the developing human brain. *Neuroimage* 202:116149. 10.1016/j.neuroimage.2019.116149 31476430 PMC6819257

[B61] Ziegler G. Dahnke R. Jancke L. Yotter R. A. May A. Gaser C. (2012). Brain structural trajectories over the adult lifespan. *Hum. Brain Mapp.* 33 2377–2389. 10.1002/hbm.21374 21898677 PMC6870331

