# Supplementary material for: Structural and Functional Trajectories of Middle Temporal Gyrus Sub-Regions During Life Span: A Potential Biomarker of Brain Development and Aging
Source: Front Aging Neurosci. 2022 Apr 27;14:799260. doi: 10.3389/fnagi.2022.799260 (PMC9094684; doi:10.3389/fnagi.2022.799260)
Supplement: Supplementary file 1 [file Data_Sheet_1.pdf]

Table S1. Subjects' information.

Groups	All	children and adolescent	young adults	middle adults	older adults	older
Subjects	160	25	48	37	22	28
Gender(male: female)	94 : 66	15:10	26:22	26:11	15:7	12:16
Age(mean (range))	7-85	7-18	19-30	31-45	46-60	61-85
Hand(right : left)	140:20	22:3	43:5	31:6	20:2	24:4

Figure S1. Sub-regions of MTG. Abbreviations: L, left; R, right; aMTG, anterior part of middle temporal gyrus (MTG); mMTG, middle part of MTG; pMTG, posterior part of MTG; and sMTG, sulcus part of MTG.

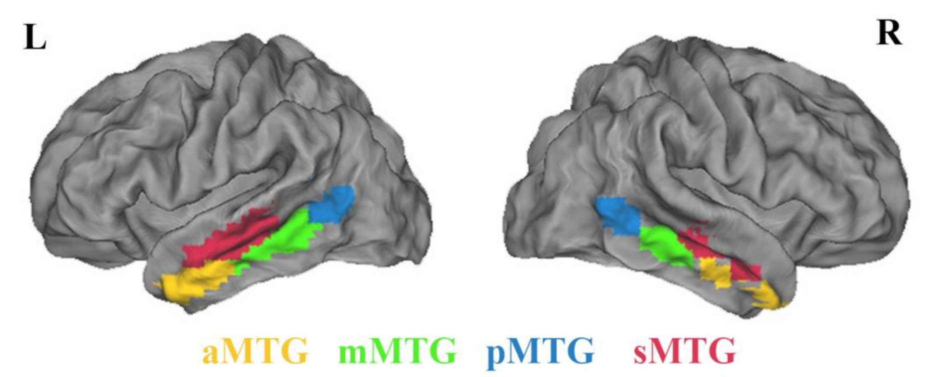

Figure S2. The prediction model is based on deep learning network. Features served as inputs for a simple 4-layer long-short term memory (LSTM) network with 40 hidden units. Each hidden unit was fully connected and defined by the equations in the right bottom.

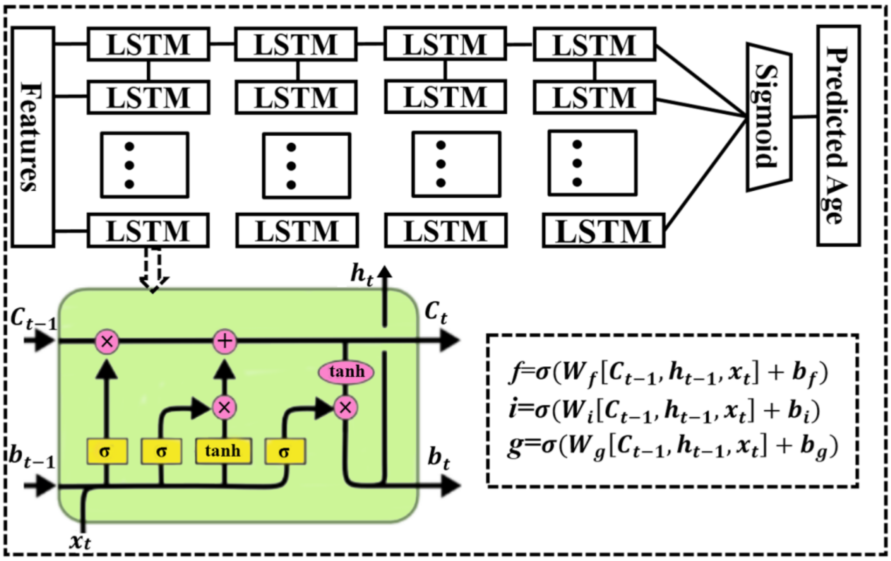
Features
LSTM
Sigmoid
Predicted Age
h_t
C_{t-1}
C_t
tanh
σ
b_{t-1}
b_t
x_t
f=$\sigma(W_f[C_{t-1}, h_{t-1}, x_t] + b_f)$
i=$\sigma(W_i[C_{t-1}, h_{t-1}, x_t] + b_i)$
g=$\sigma(W_g[C_{t-1}, h_{t-1}, x_t] + b_g)$
